# Supplementary material for: Rapid Eocene diversification of spiny plants in subtropical woodlands of central Tibet
Source: Nat Commun. 2022 Jul 1;13:3787. doi: 10.1038/s41467-022-31512-z (PMC9249787; doi:10.1038/s41467-022-31512-z)
Supplement: Supplementary file 1 — Supplementary Information [file 41467_2022_31512_MOESM1_ESM.docx]

**Supplementary information for ‘****Rapid Eocene diversification of spiny plants in subtropical woodlands of central Tibet’**

**This PDF file includes:**

Supplementary Figure 1 **│**Two fossil sites in this study from the central Tibetan Plateau.

Supplementary Figure 2 **│**Fossil spines morphotype Ⅰ.

Supplementary Figure 3 **│**Fossil spines morphotype Ⅱ.

Supplementary Figure 4 **│**Fossil spines morphotype Ⅲ.

Supplementary Figure 5 **│**Fossil spines morphotype Ⅳ.

Supplementary Figure 6 **│**Fossil spines morphotype Ⅴ.

Supplementary Figure 7 **│**Fossil spines morphotype Ⅵ.

Supplementary Figure 8 **│**Fossil spines morphotype Ⅶ.

Supplementary Figure 9 **│**Leaf morphotypes of the Dayu flora Ⅰ.

Supplementary Figure 10 **│**Leaf morphotypes of the Dayu flora Ⅱ.

Supplementary Figure 11 **│**Six morphotypes of herbaceous plant macrofossils from the Dayu section, central Tibetan Plateau.

Supplementary Figure 12 **│**Main morphotypes of phytoliths extracted from the sedimentary of the Dayu section, central Tibetan Plateau.

Supplementary Figure 13 **│**Composition of phytoliths in different layers of the Dayu and Jianglang sections.

Supplementary Figure 14 **│**Climate modelling, topography, vegetation types and soil moisture conditions.

Supplementary Figure 15 **│**Spines observed in living palms.

Supplementary Table 1 **│**Palaeoclimate conditions of the Dayu and Jianglang leaf assemblages using Climate-Leaf Analysis Multivariate Program (CLAMP).

Supplementary Table 2 **│**Climate-Leaf Analysis Multivariate Program (CLAMP) scoresheet for Dayu (~39 Ma).

Supplementary Table 3 **│**Climate-Leaf Analysis Multivariate Program (CLAMP) scoresheet for Jianglang (~47 Ma).

Supplementary Table 4 │Phytolith statistics of the Dayu and Jianglang sections.

Supplementary Table 5 **│**Modern affinities of elements of the Dayu flora that potentially have spines.

Supplementary Note 1 **│**Detailed morphological description of each morphotype of fossil spines.

Supplementary Note 2 **│**Palaeoelevation reconstruction of Dayu at ~39 Ma.

Supplementary Note 3 **│**Reconstructed vegetation using climate modelling.

Supplementary References

**Supplementary Figure 1 │Two fossil sites in this study from the central Tibetan Plateau.** A and B, Dayu site; C, Xiede site. Note that vehicles between two pointed layers serve as a scale.


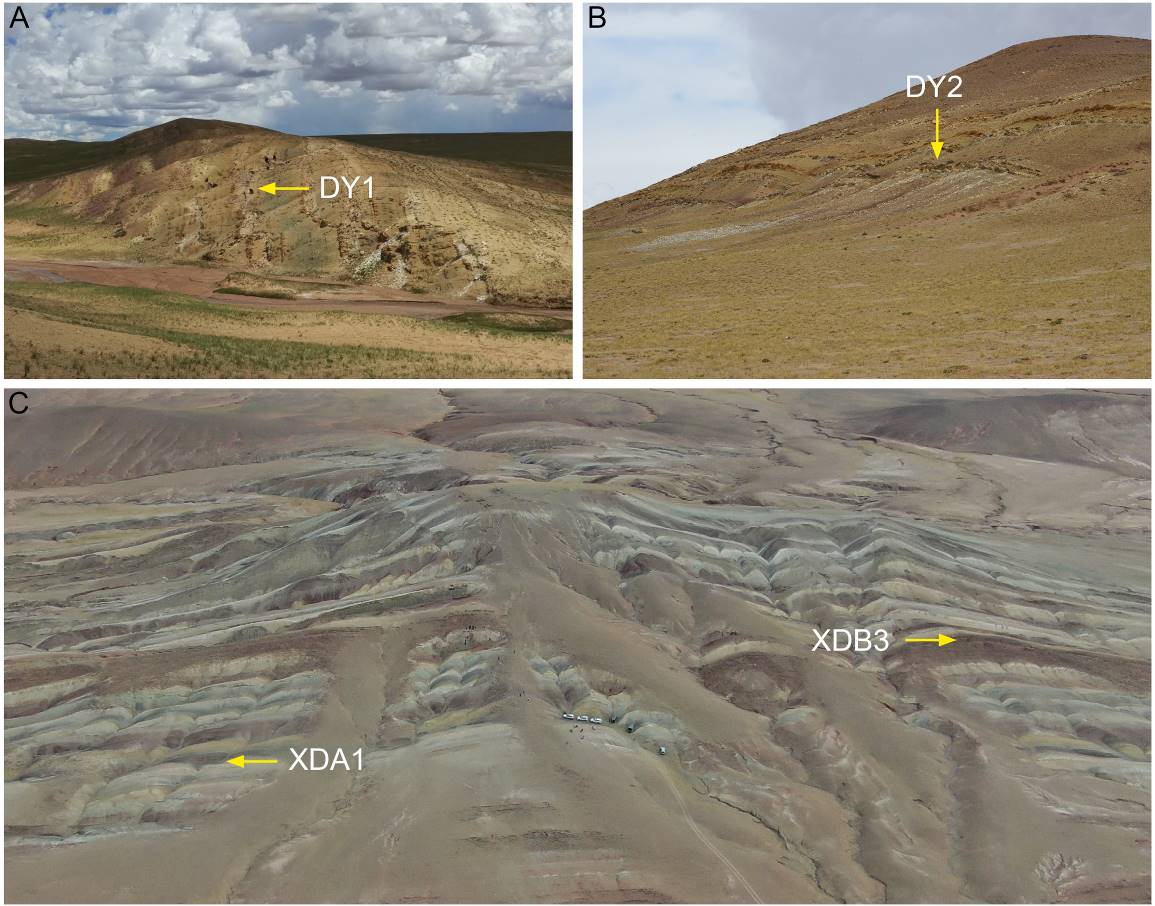


**Supplementary Figure 2 │Fossil spines morphotype Ⅰ.** (Scale bars = 1 cm)


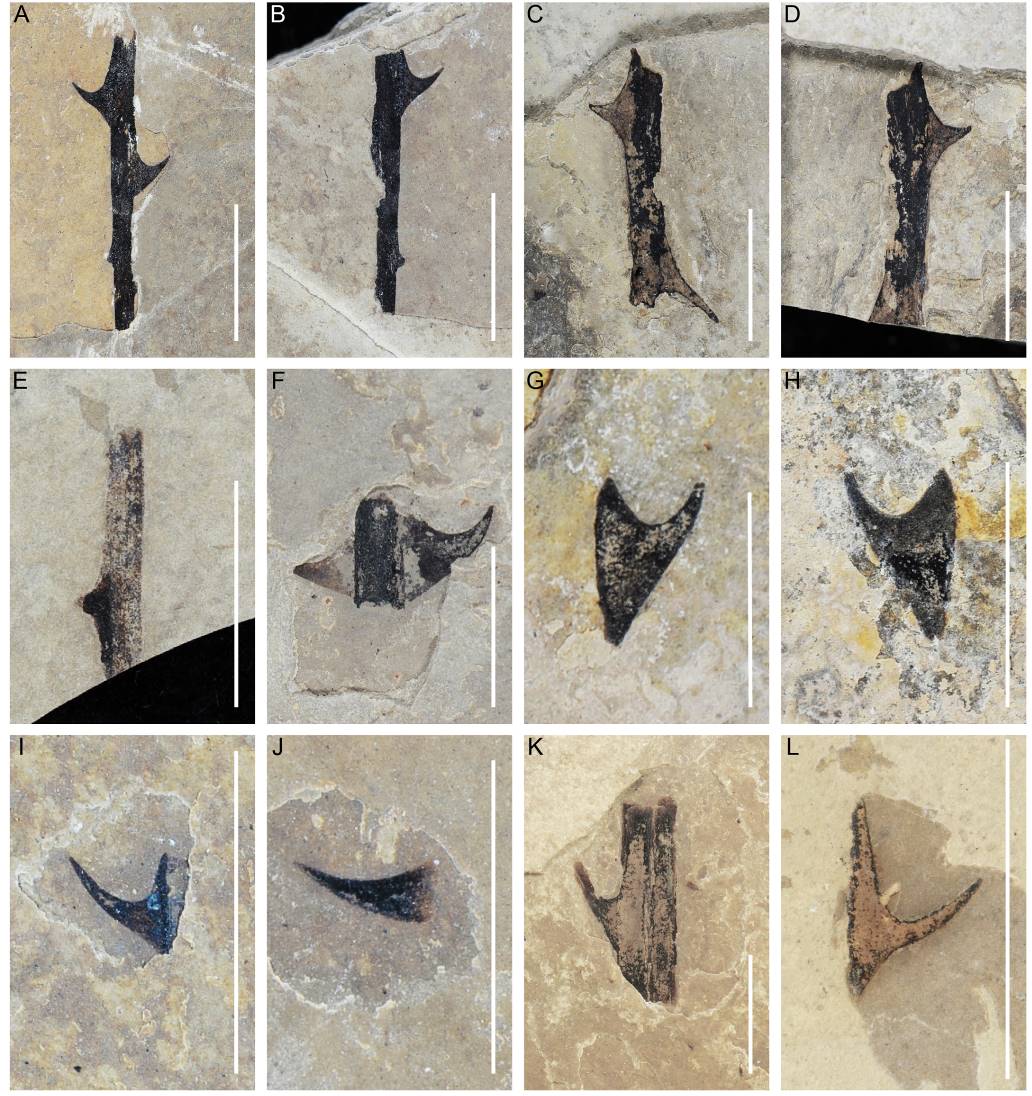


Specimen numbers: A, XZXDA1-0004a; B, XZXDA1-0004b; C, XZXDA1-0010b; D, XZXDA1-0010a; E, XZXDA1-0002; F, XZXDA1-0001; G, XZXDA1-0011a; H, XZXDA1-0011b; I, XZXDA1-0007; J, XZXDA1-0003; K, XZXDB3-0003; L, XZXDA2-0454.

**Supplementary Figure 3 │Fossil spines morphotype Ⅱ.** (Scale bars = 1 cm)


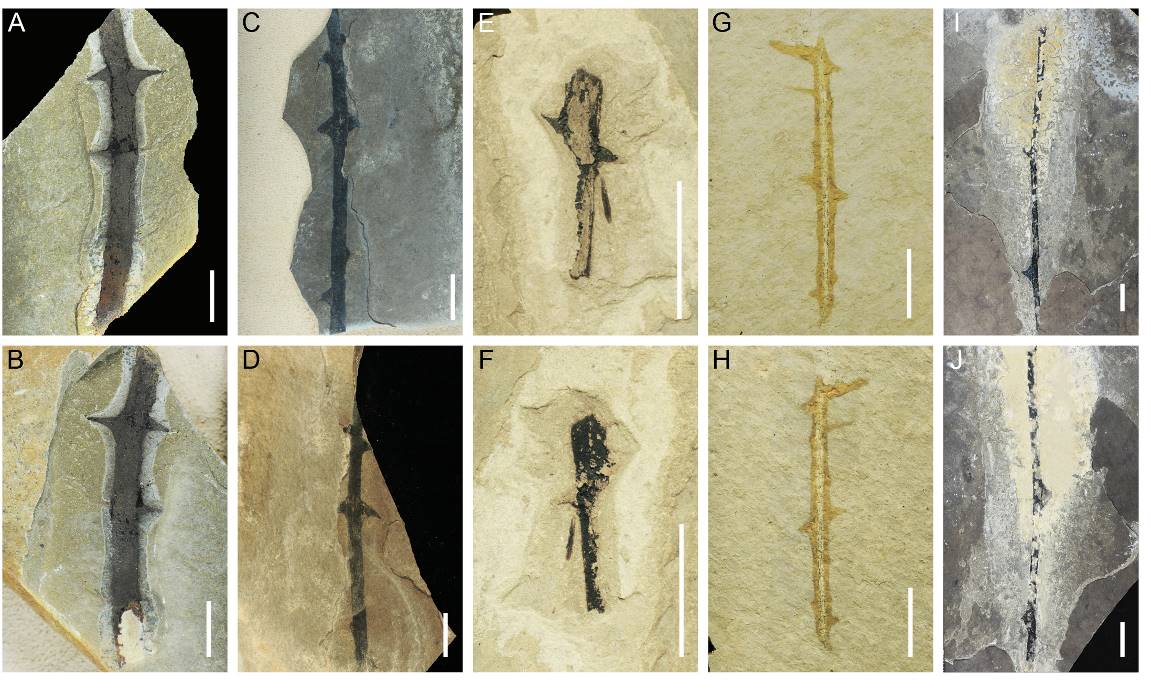


Specimen numbers: A, XZDY2-0020a; B, XZDY2-0020b; C, XZDY2-0217a; D, XZDY2-0217b; E, XZDY2-0158a; F, XZDY2-0158b; G, XZDY2-0216a; H, XZDY2-0216b; I, XZXDA1-0008a; J, XZXDA1-0008b.

**Supplementary Figure 4 │Fossil spines morphotype Ⅲ.** (Scale bar = 1 cm)


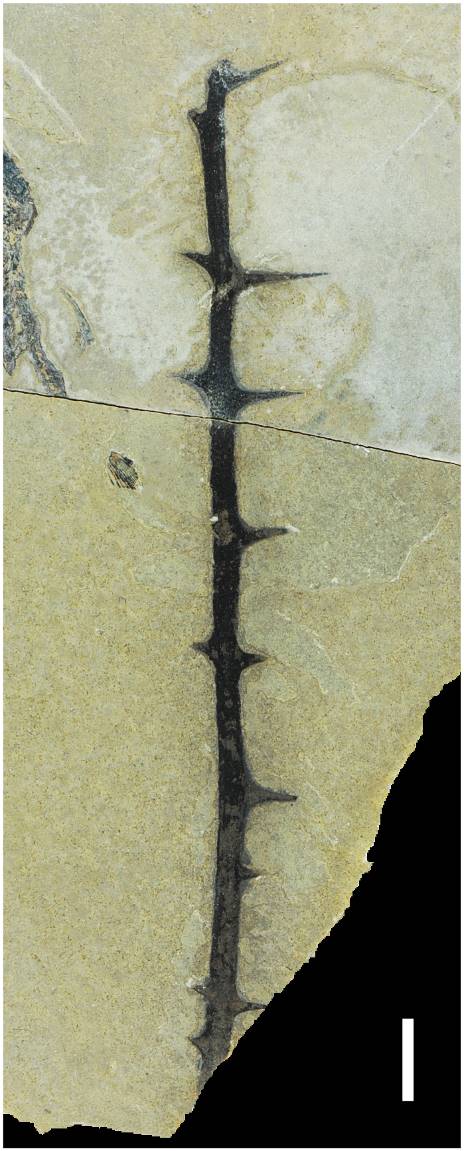


Specimen number: XZDY1-0061.

**Supplementary Figure 5 │Fossil spines morphotype Ⅳ.** (Scale bars = 1 cm)


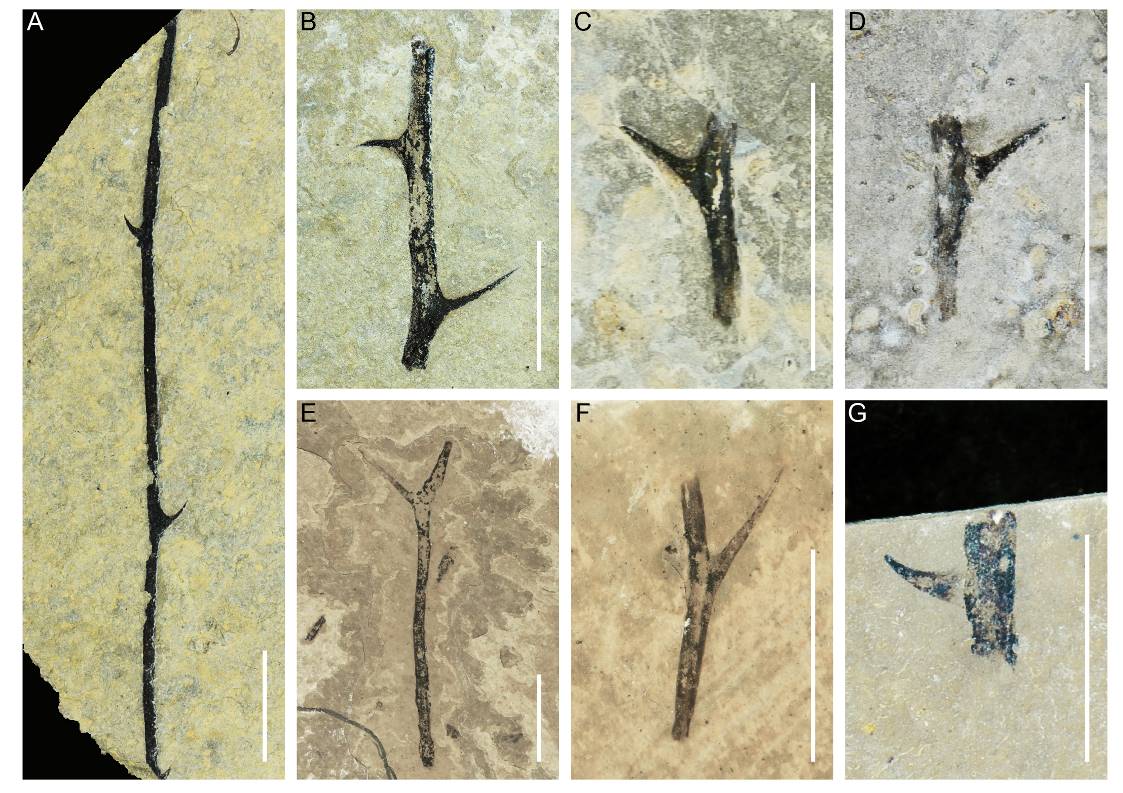


Specimen numbers: A, XZDY1-0381; B, XZDY2-0221; C, XZXDA1-0013a; D, XZXDA1-0013b; E, XZXDB3-0005; F, XZXDB3-0004; G, XZXDA1-0006.

**Supplementary Figure 6 │Fossil spines morphotype Ⅴ.** (Scale bars = 1 cm)


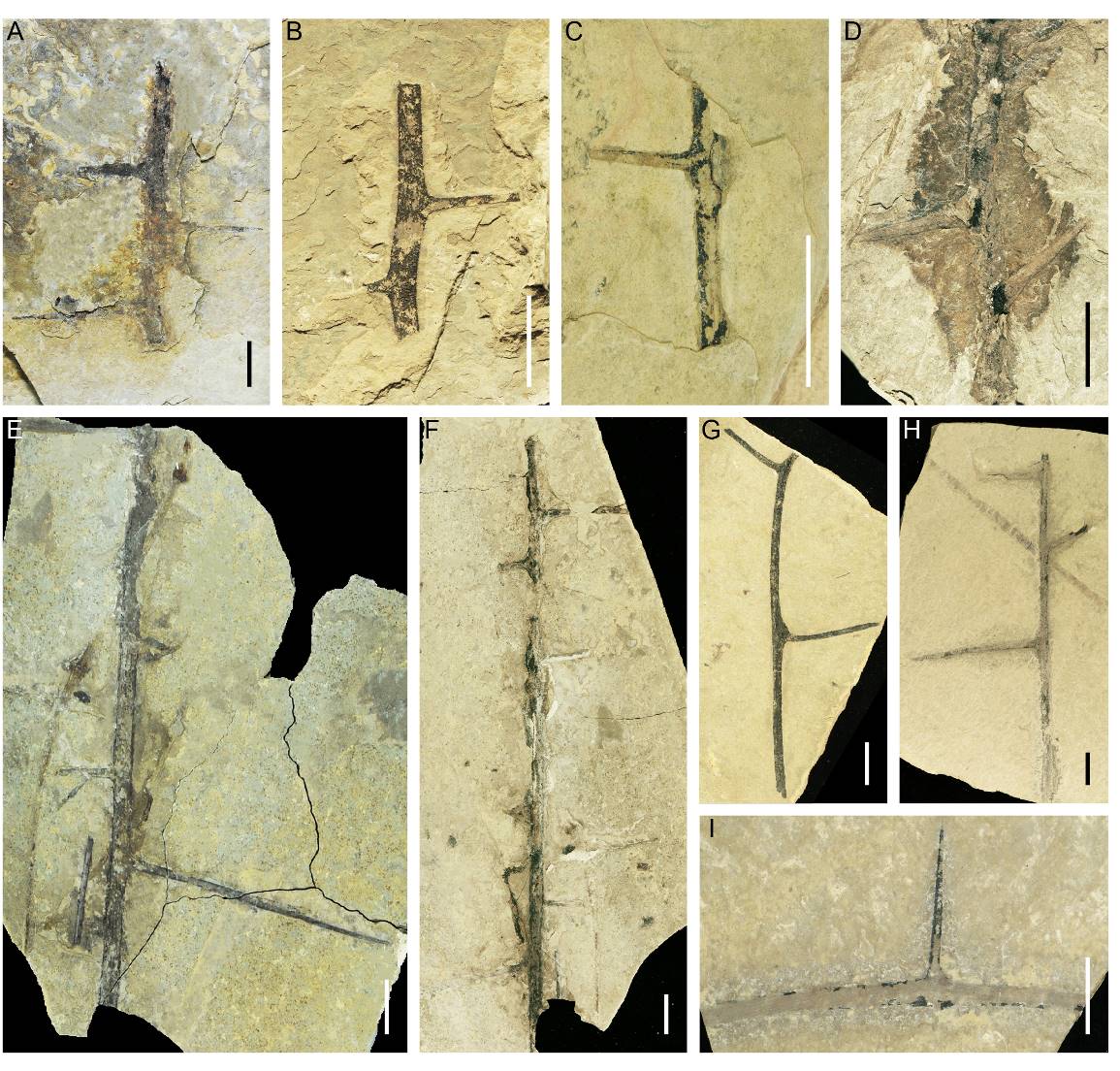


Specimen numbers: A, XZXDA1-0014; B, XZDY2-0220; C, XZDY2-0055; D, XZDY1-0362; E, XZDY1-0336; F, XZDY1-0339; G, XZDY2-0374; H, XZDY2-0213; I, XZXDB3-0002.

**Supplementary Figure 7 │Fossil spines morphotype Ⅵ.** (Scale bar = 1 cm)


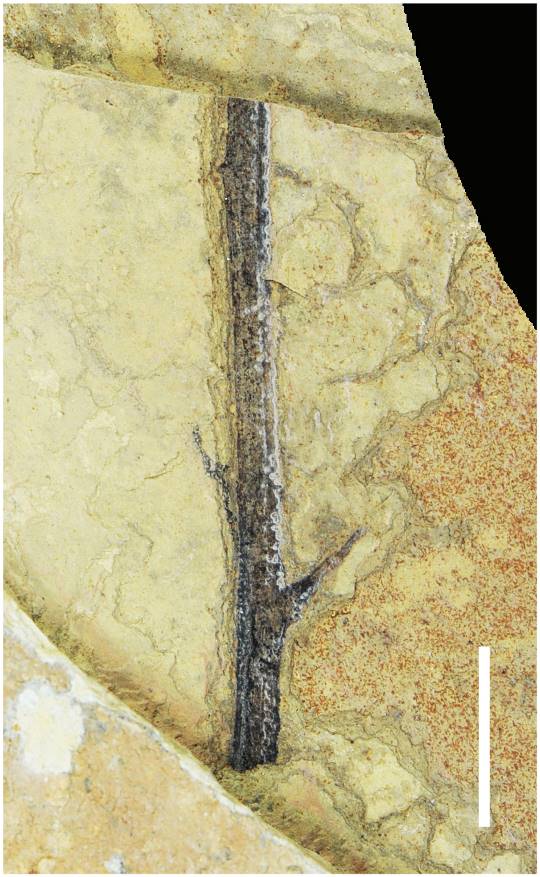


Specimen number: XZDY1-0118.

**Supplementary Figure 8 │Fossil spines morphotype Ⅶ.** (Scale bars = 1 cm)


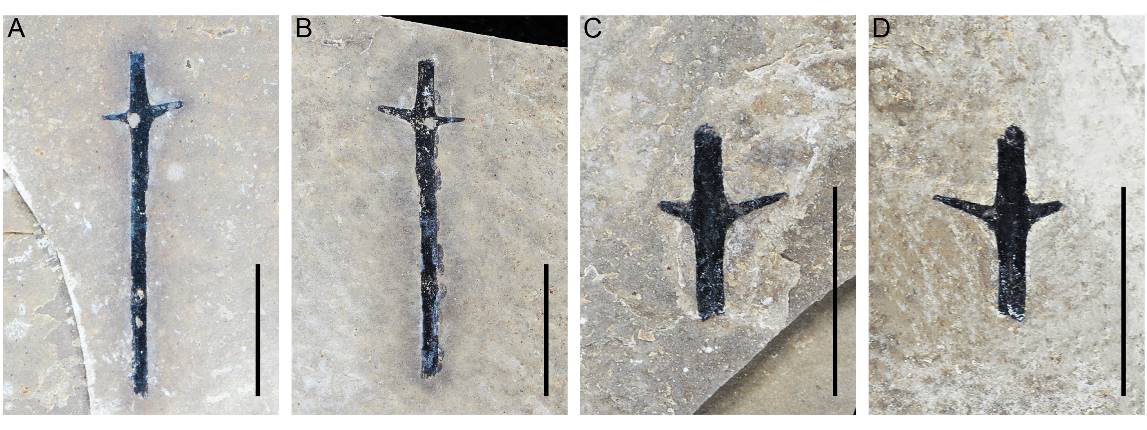


Specimen numbers: A, XZXDA1-0005a; B, XZXDA1-0005b; C, XZXDA1-0012a; D, XZXDA1-0012b.

**Supplementary Figure 9 │Leaf morphotypes of the Dayu flora Ⅰ.** (Scale bars = 1 cm)


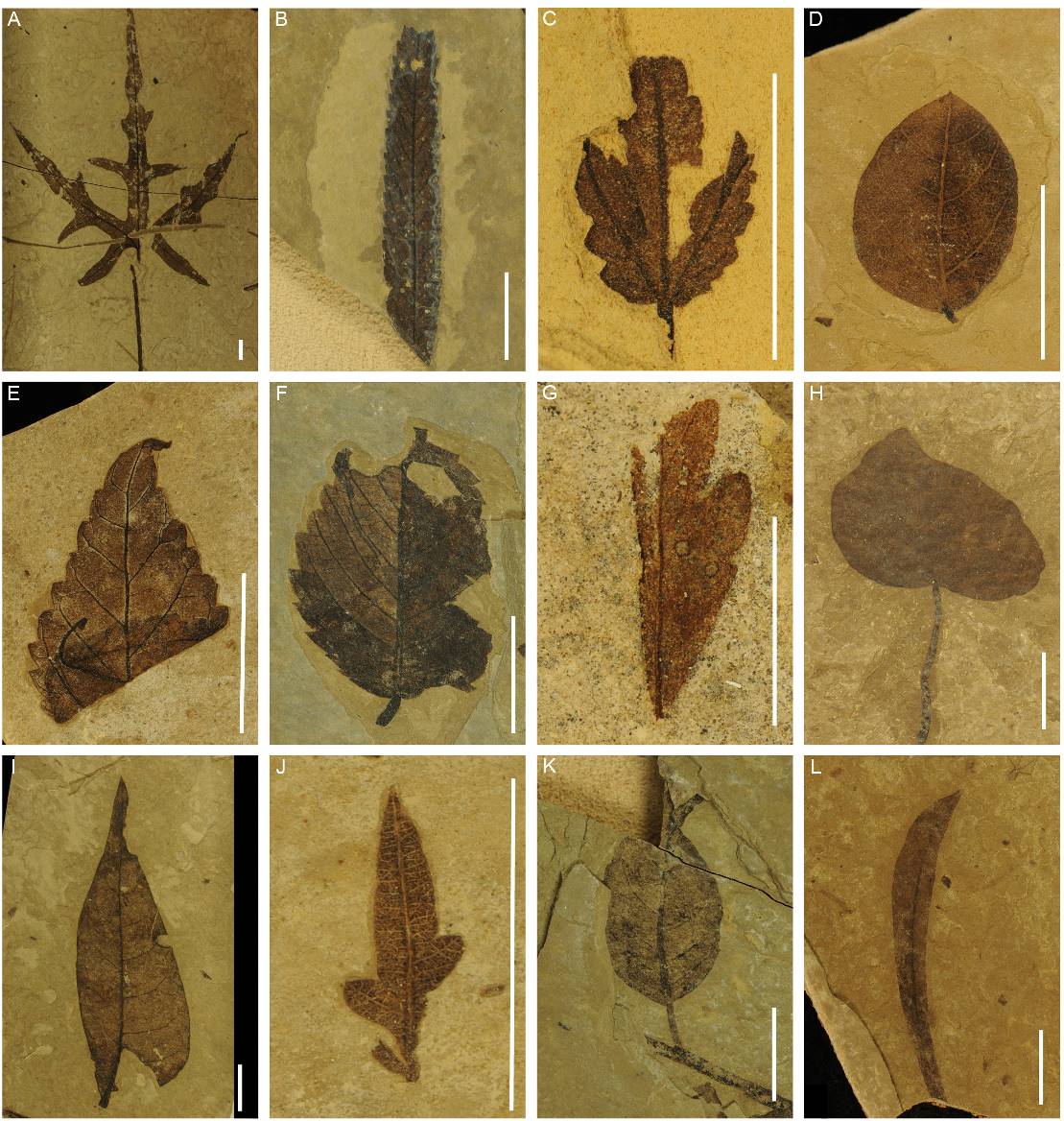


A, M01, Malvaceae (morphytype 1), XZDY2-037; B, M02, *Cedrelospermum*, XZDY1-0060; C, M03, Cannabacea, XZDY1-0097e; D, M04, *Desmodium*, XZDY1-0231; E, M05, Rosales (Cannabaceae or Urticaceae), XZDY1-0256; F, M06, *Ulmus*, XZDY2-0072; G, M07, Small and lobed leaf, XZDY1-0240; H, M08, Menispermaceae, XZDY3-0001; I, M09, *Ailanthus*, XZDY2-0372; J, M10, Anacardiaceae, XZDY1-0371 K, XZDY2-0022; L, M12, Myrtaceae, XZDY3-0031.

**Supplementary Figure 10 │Leaf morphotypes of the Dayu flora Ⅱ.** (Scale bars = 1 cm)


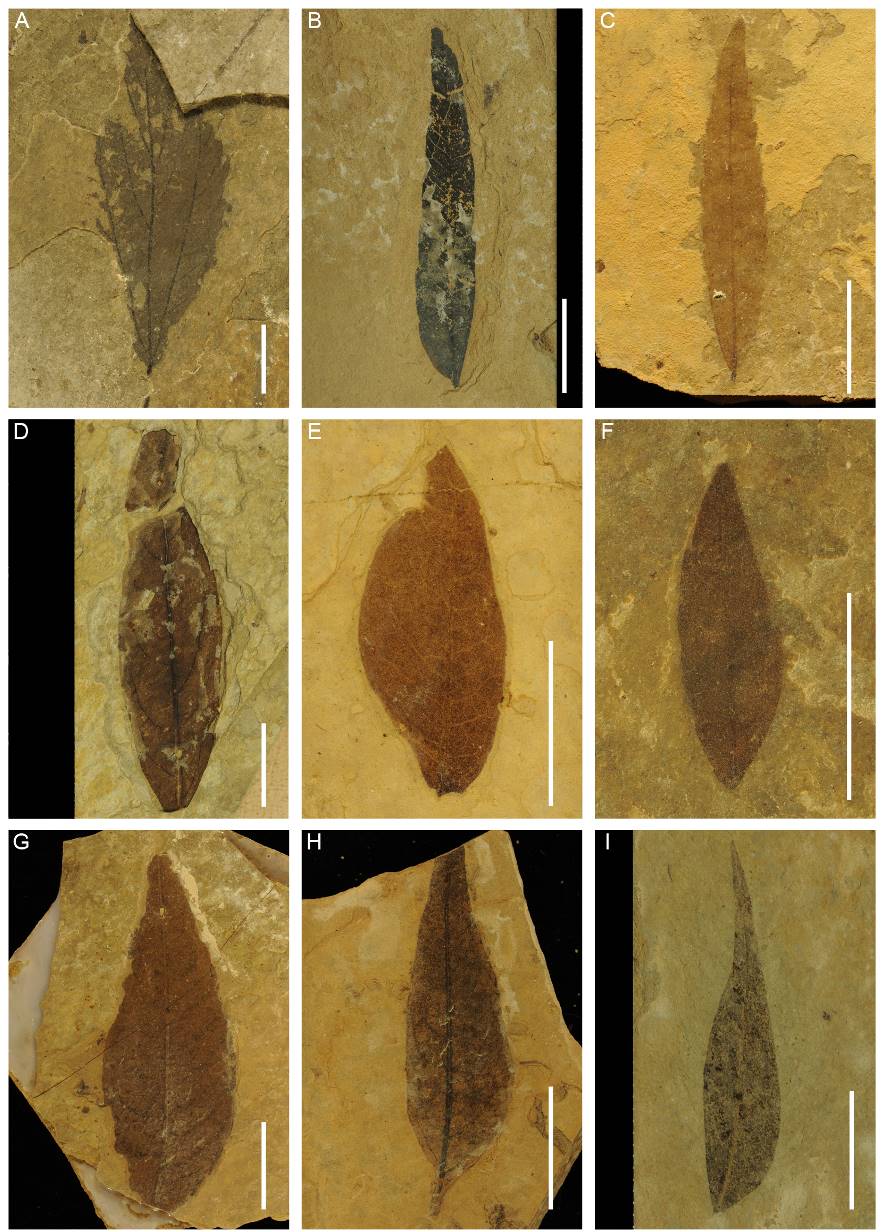


A, M13, Malvaceae (morphotype 2), XZDY2-0146; B, M14, *Syzygium*, XZDY2-0045; C, M15, Leaf elongate with regular marginal teeth, XZDY2-0145; D, M16, Leaf base acute with margin entire, XZDY2-0058; E, M17, leaflet of Fabaceae (morphotype 1), XZDY1-0302; F, M18, Small leaf with few teeth, XZDY1-0085; G, M19, Small and elliptic leaf with entire margin, XZDY1-0275; H, M20, Leaflet of Fabaceae (morphotype2), XZDY1-0150; I, M21, *Pistacia*, XZDY2-0029. We also score three fossil leaves from Wu et al.^1^, these are: WuFX-FIG3f: Small leaf with round apex and acute base; WuFX-FIG3j: Araliaceae; WuFX-FIG3j: *Handeliodendron*.

**Supplementary Figure 11 │Six morphotypes of herbaceous plant macrofossils from the Dayu section, central Tibetan Plateau.** A, Leaves clustered at the top of the stem; B, sympodial branching; C, Leaves are clustered at the base of the stem. The stem has obvious nodes that are closely spaced; D, Leaf 2–2.5 cm wide, with parallel veins; E. The stem has swollen nodes; F, Fascicled and slender leaf with a distinct main vein. (Scale bars = 1 cm)


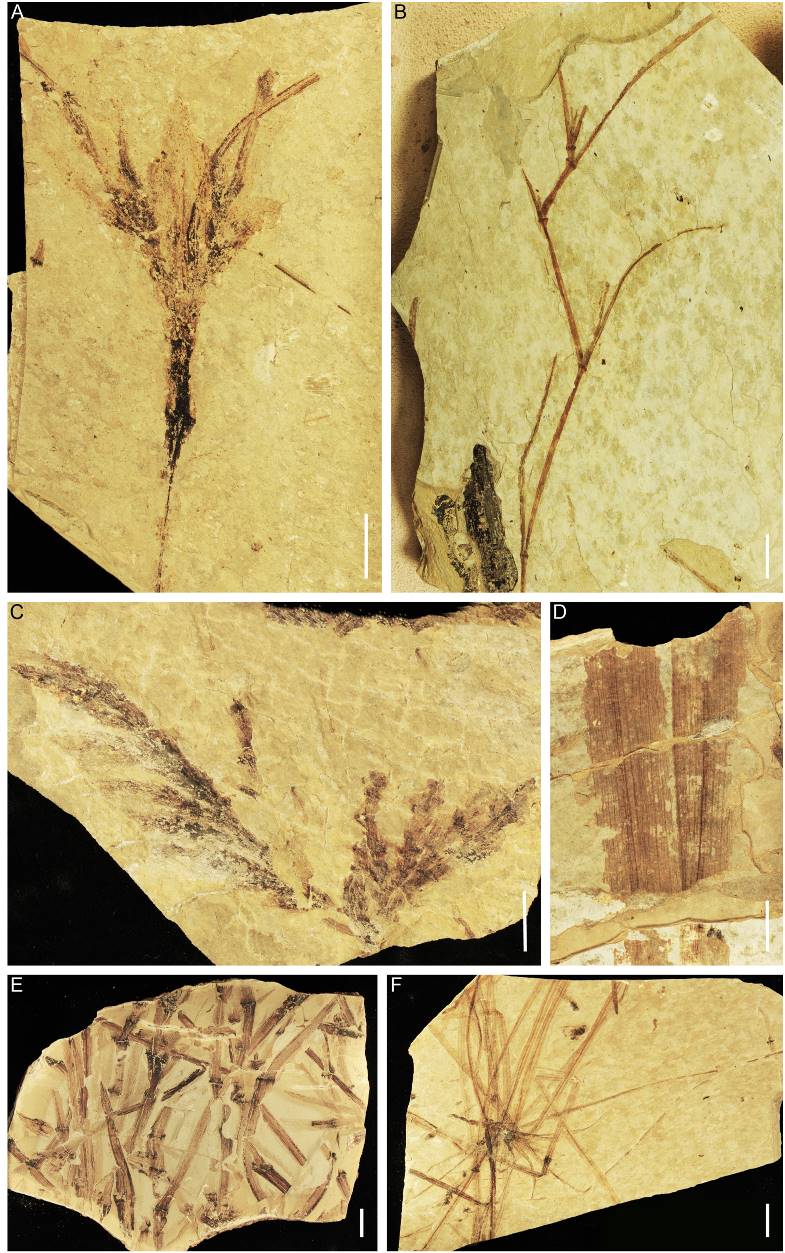


Specimen numbers: A, XZDY1-0203A; B, XZDY2-0003; C, XZDY2-0248A; D, XZDY1-0228A; E, XZDY2-0305B; F, XZDY2-0161B.

**Supplementary Figure 12 │Main morphotypes of phytoliths extracted from the sediments of the Dayu section, central Tibetan Plateau.** A, B, True saddle, with a pair of short ridges, both are raised in a saddle shape and derived from Chloridoideae, which today are C_4_ plants; C-F, Rondel, looks like a circular table with an oval bottom, a type unique to Pooideae; E, Rondel with saddle top; G, H, I, Bulliform, shape resembles an expanded folding fan, derived from plant motor cells; H potentially from Bambusoideae^2^; J, undetermined, possible a highly etched motor cell derived from Poaceae; K, Square; L, Elongate sinuate; M, Elongate dentate; N, O, Blocky, mainly derived from woody plants. P, undetermined, possible a collapsed saddle or a broken bilobate; Q, undetermined; R, Sponge spicule; S, T, Diatom. For phytolith analysis, we performed 3 replicate experiments with consistent results. (Scale bars = 10 µm)


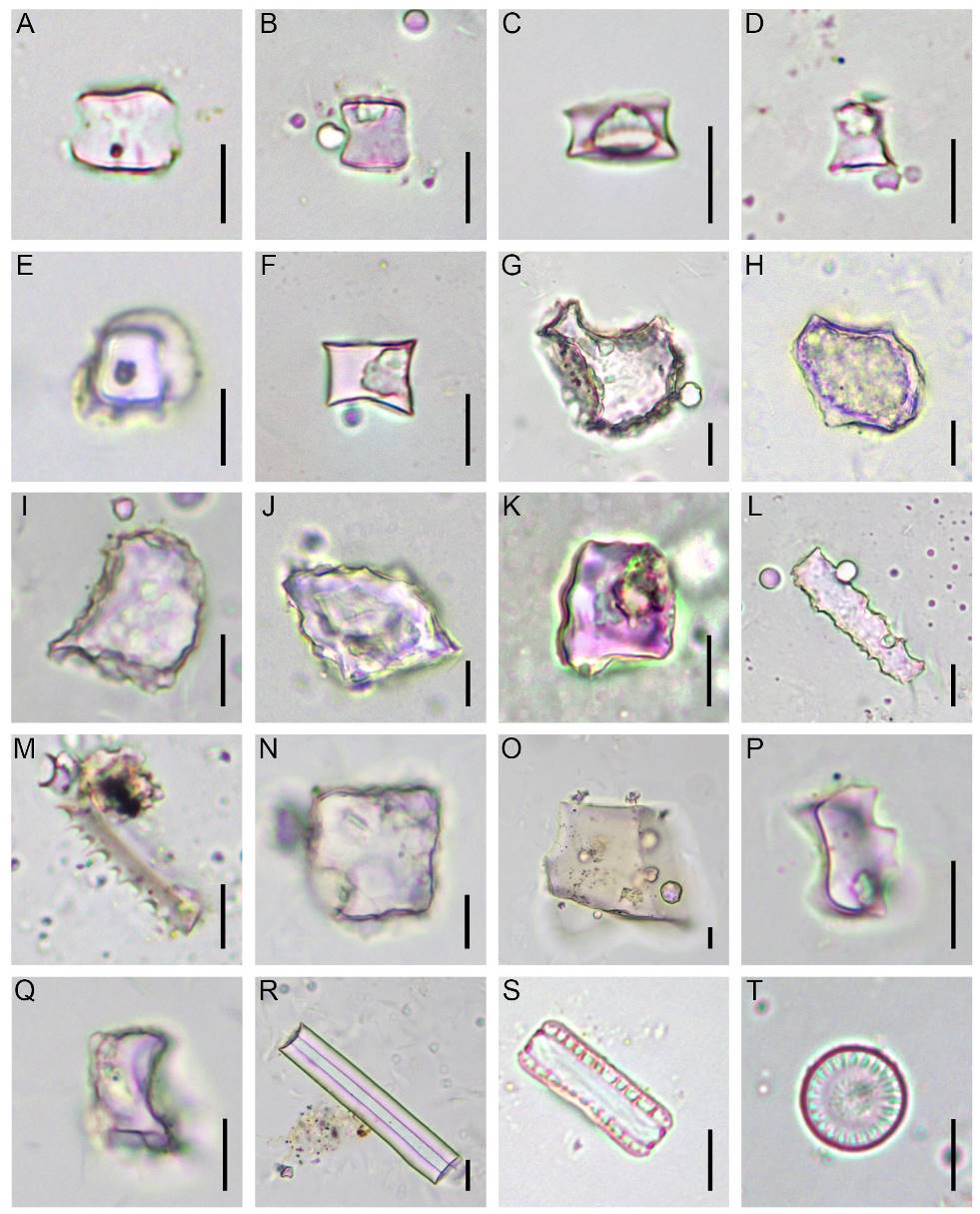


Specimen numbers: XZDY0049, XZDY0050, XZDY0051.

**Supplementary Figure 13 │Composition of phytoliths in different layers of the Dayu**^3^ **and Jianglang sections**^4^**.** Pie chart representing the percentage of grass phytoliths (orange) to woody plant phytoliths (blue).


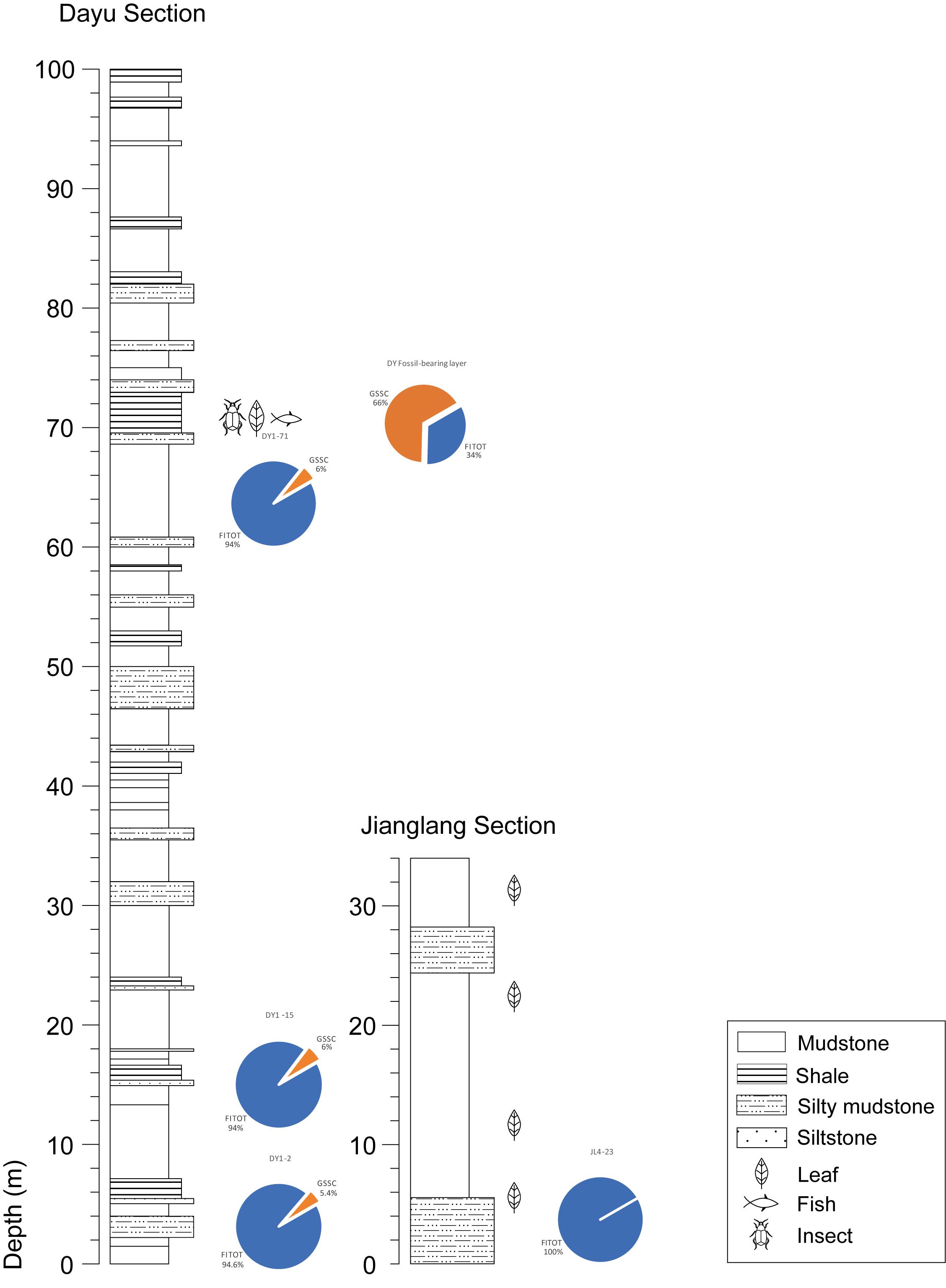


**Supplementary Figure 14 │Climate modelling, topography, vegetation types and soil moisture conditions.** A, Topography of the Tibetan region used in the modelling with a central valley floor set at 2.5 km bounded by twin mountain ranges set at 5 km. B, Predicted vegetation types using Triffid with Priabonian boundary conditions. The black rectangle highlights the Central Tibetan Valley. C, Precipitation and soil moisture conditions for the Lunpola region.


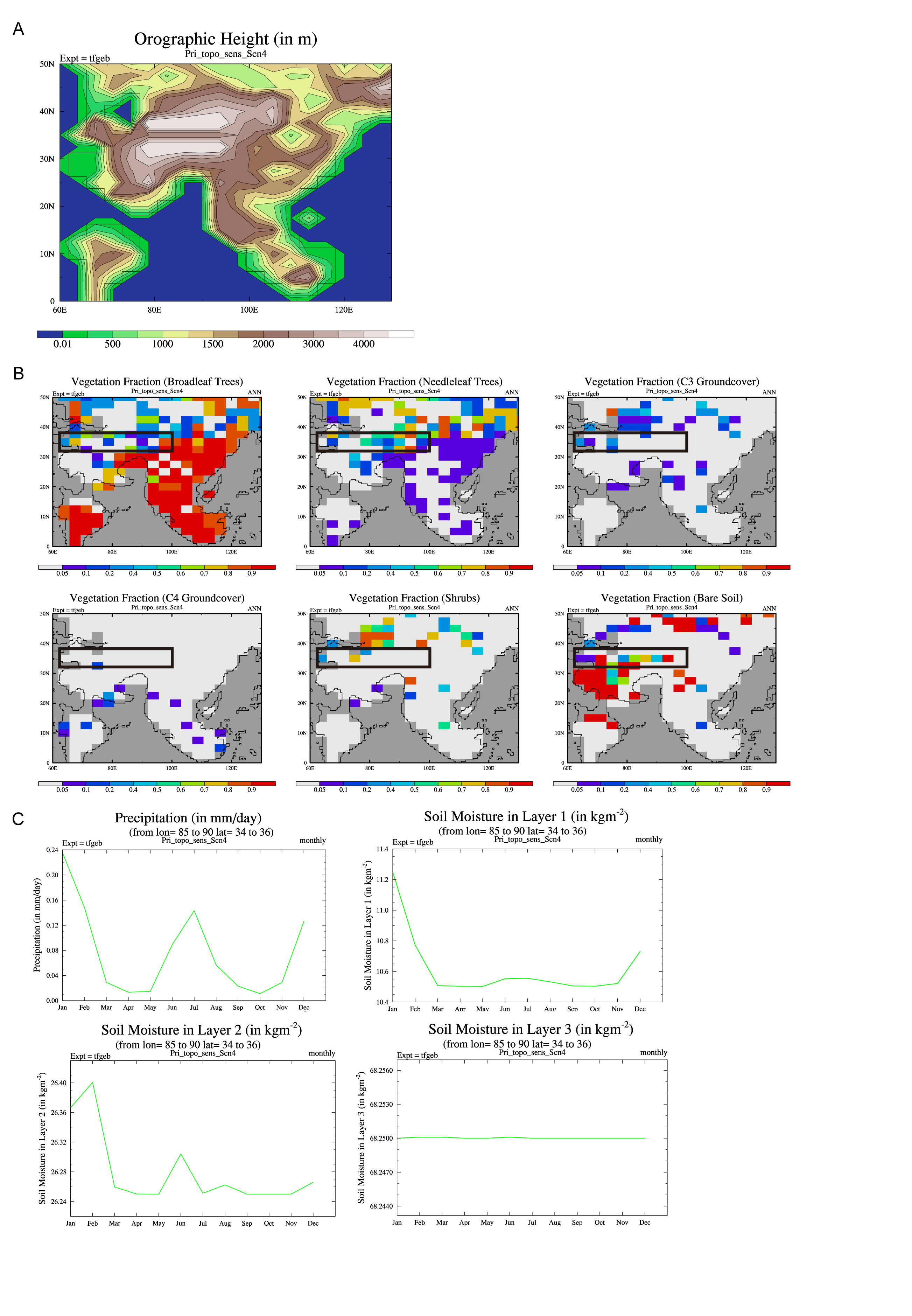


**Supplementary Figure 15 │Spines observed in living palms.** A, *Corypha utan*; B, *Hyphaene thebaica*; C, *Copernicia prunifera*; D, *Elaeis oleifera*; E, *Corypha umbraculifera*; F, *Licuala peltate*; G, *Daemonorops margaritae*; H, *Livistona cochinchinensis*; I, *Butia yatay*. Photos were taken in Xishuangbanna Tropical Botanical Garden. (Scale bars = 1 cm)


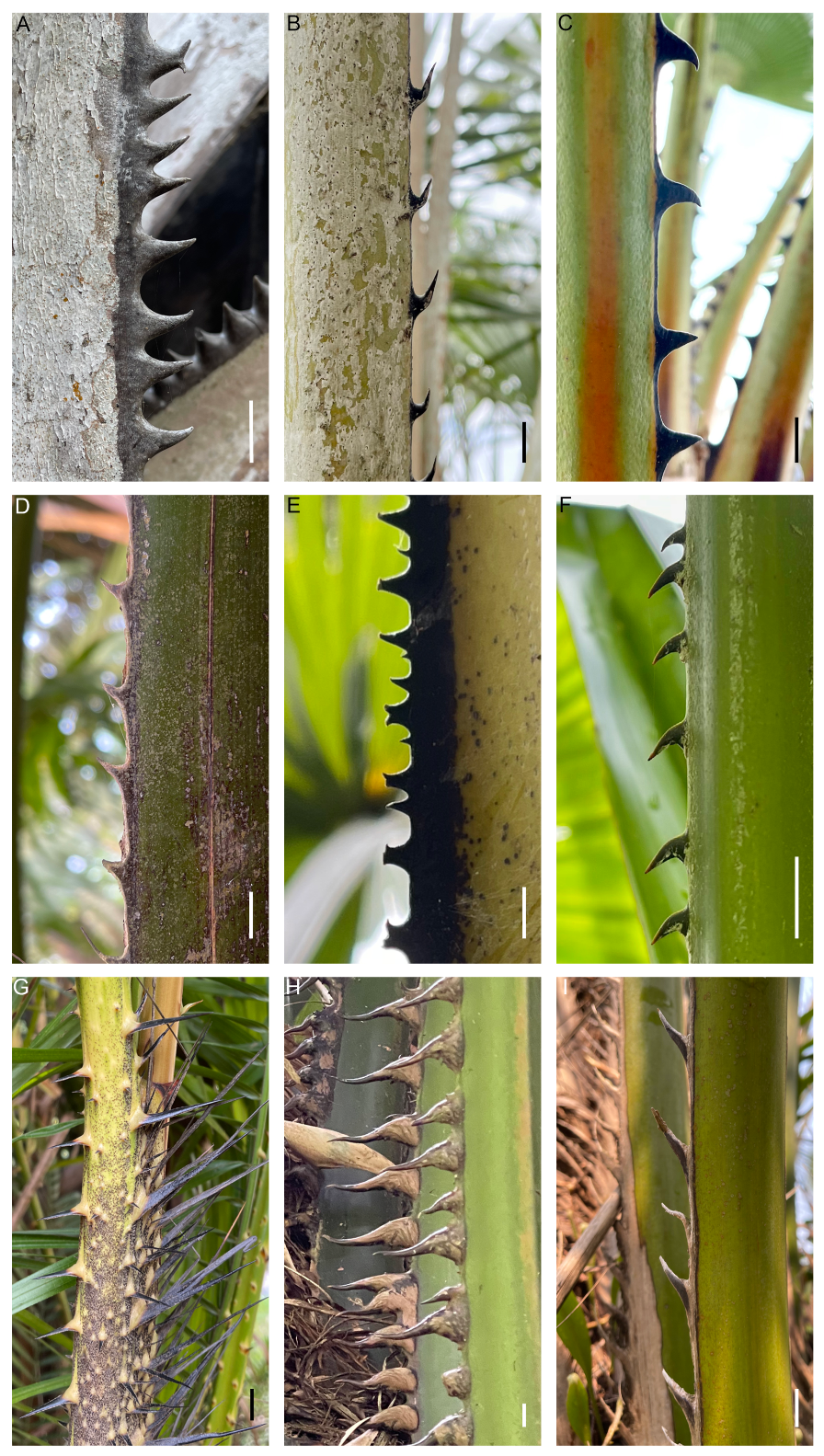


**Supplementary Table 1 │Palaeoclimate conditions of the Dayu and Jianglang leaf assemblages using Climate-Leaf Analysis Multivariate Program (CLAMP).**

| Climate Variables | Dayu | Jianglang | Standard Deviation |
| --- | --- | --- | --- |
| Mean annual dry bulb temperature (MAT) (°C) | 15.6 | 19.3 | 2.4 |
| Mean annual wet bulb temperature (MATw) (°C) | 10.2 | 13.4 | 2.5 |
| Warm month mean dry bulb temp. (WMMT) (°C) | 27.2 | 28.8 | 2.9 |
| Cold month mean dry bulb (CMMT) (°C) | 3.8 | 8.0 | 3.5 |
| Growing Season Length (months) | 9.5 | 11.2 | 1.1 |
| Growing Season Precipitation (cm) | 178 | 208 | 64 |
| Mean Monthly Growing Season Precipitation (cm) | 18.4 | 20.8 | 6.5 |
| Precipitation in the three wettest months (cm) | 85.5 | 100 | 40 |
| Precipitation in the three driest months (cm) | 18.9 | 16.3 | 9.8 |
| Specific Humidity (g/kg) | 7.0 | 8.8 | 1.8 |
| Moist Enthalpy (kJ/kg) | 316 | 327 | 8.4 |
| Mean Annual Vapour Pressure Deficit (hPa) | 9.1 | 10.8 | 2.4 |
| Mean Winter Vapour Pressure Deficit (hPa) | 4.3 | 5.8 | 1.5 |
| Mean Spring Vapour Pressure Deficit (hPa) | 7.9 | 10.5 | 3.9 |
| Mean Summer Vapour Pressure Deficit (hPa) | 13.7 | 15.1 | 3.5 |
| Mean Autumn Vapour Pressure Deficit (hPa) | 9.7 | 11.0 | 2.0 |
| Compensated Thermicity Index (°C) | 279 | 401 | 75 |
| Minimum Temperature of Warmest Month (°C) | 22.9 | 22.3 | 2.9 |
| Maximum Temperature of Coldest Month (°C) | 10.2 | 15.0 | 3.5 |
| Growing Degree Days > 0°C × 10^-3^ | 64.6 | 84.6 | 11.8 |
| Growing Degree Days > 5°C × 10^-3^ | 69.8 | 87.0 | 10.6 |
| Annual Potential Evapotranspiration (mm/month) | 116.7 | 131.8 | 16.6 |
| Potential Evapotranspiration in Warmest 3 Months (mm/month) | 154.9 | 161.1 | 24.5 |
| Potential Evapotranspiration in Coldest 3 Months (mm/month) | 32.9 | 48.6 | 14.0 |

**Supplementary Table 2 │Climate-Leaf Analysis Multivariate Program (CLAMP) scoresheet for Dayu (~39 Ma).** Scores of morphotypes 1–21 are from Supplementary Figures 9 and 10, scores of morphotypes WuFX-FIG3f, WuFX-FIG3i and WuFX-FIG3j are from Wu et al.^1^. Colours of the heading line represent different leaf traits. Colours of scoring rows represent different leaf morphotypes.

| **Species  Number** | **Species / Morphotypes** | **Lamina** | | **Margin Character States** | | | | | | | | | | **Size Character States** | | | | | | | | | **Apex Character States** | | | | **Base Character States** | | | **Length to Width Character States** | | | | | **Shape Character States** | | |
| --- | --- | --- | --- | --- | --- | --- | --- | --- | --- | --- | --- | --- | --- | --- | --- | --- | --- | --- | --- | --- | --- | --- | --- | --- | --- | --- | --- | --- | --- | --- | --- | --- | --- | --- | --- | --- | --- |
|  |  | **Unlobed** | **Lobed** | **No Teeth** | **Teeth** | **Teeth Regular** | **Teeth Irregular** | **Teeth Close** | **Teeth Distant** | **Teeth Round** | **Teeth Acute** | **Teeth Compound** | **Compound<50%** | **Nanophyll** | **Leptophyll I** | **Leptophyll II** | **Microphyll I** | **Microphyll II** | **Microphyll III** | **Mesophyll I** | **Mesophyll II** | **Mesophyll III** | **Emarginate** | **Round** | **Acute** | **Attenuate** | **Cordate** | **Round** | **Acute** | **L:W <1:1** | **L:W 1-2:1** | **L:W 2-3:1** | **L:W 3-4:1** | **L:W >4:1** | **Obovate** | **Elliptic** | **Ovate** |
| 1 | **M01** |  | **1** | **1** |  |  |  |  |  |  |  |  |  |  |  |  | **1** | **1** |  |  |  |  |  |  |  | **1** | **1** |  |  |  | **1** |  |  |  |  | **1** |  |
| 2 | **M02** | **1** |  |  | **1** | **1** |  | **1** |  | **1** |  |  |  |  |  |  |  | **1** |  |  |  |  |  |  |  |  |  |  |  |  |  |  |  | **1** |  | **1** |  |
| 3 | **M03** | **1** |  |  | **1** | **1** |  | **1** |  | **1** |  |  |  |  |  | **1** |  |  |  |  |  |  |  |  | **1** |  |  | **1** |  |  | **1** |  |  |  |  | **1** |  |
| 4 | **M04** | **1** |  | **1** |  |  |  |  |  |  |  |  |  |  |  |  | **1** |  |  |  |  |  |  |  | **1** |  |  | **1** |  |  | **1** |  |  |  |  | **1** |  |
| 5 | **M05** | **1** |  |  | **1** | **1** |  | **1** |  | **1** | **1** |  |  |  |  |  |  |  | **1** |  |  |  |  |  |  | **1** |  | **1** |  |  | **1** |  |  |  |  |  | **1** |
| 6 | **M06** | **1** |  |  | **1** |  |  |  | **1** |  | **1** |  |  |  |  |  |  | **1** |  |  |  |  |  |  |  |  | **1** |  |  |  | **1** |  |  |  |  | **1** |  |
| 7 | **M07** |  | **1** | **1** |  |  |  |  |  |  |  |  |  |  | **1** |  |  |  |  |  |  |  |  | **1** |  |  |  |  | **1** |  | **1** |  |  |  |  | **1** |  |
| 8 | **M08** | **1** |  | **1** |  |  |  |  |  |  |  |  |  |  |  |  |  | **1** |  |  |  |  |  | **1** |  |  | **1** |  |  | **1** |  |  |  |  |  | **1** |  |
| 9 | **M09** | **1** |  | **1** |  |  |  |  |  |  |  |  |  |  |  |  |  |  | **1** |  |  |  |  |  |  | **1** |  |  | **1** |  |  |  | **1** |  |  |  | **1** |
| 10 | **M10** |  | **1** | **1** |  |  |  |  |  |  |  |  |  | **1** | **1** |  |  |  |  |  |  |  |  |  | **1** |  |  |  | **1** |  |  | **1** |  |  |  | **1** |  |
| 11 | **M11** | **1** |  |  | **1** | **1** |  |  | **1** | **1** |  |  |  |  |  |  |  | **1** |  |  |  |  |  |  |  | **1** |  |  | **1** |  | **1** |  |  |  |  | **1** |  |
| 12 | **M12** | **1** |  | **1** |  |  |  |  |  |  |  |  |  |  |  |  |  | **1** |  |  |  |  |  |  |  | **1** |  |  | **1** |  |  |  |  | **1** |  |  | **1** |
| 13 | **M13** | **1** |  |  | **1** | **1** |  | **1** |  | **1** |  |  |  |  |  |  |  | **1** |  |  |  |  |  |  |  | **1** |  |  | **1** |  |  |  | **1** |  |  | **1** |  |
| 14 | **M14** | **1** |  |  | **1** | **1** |  |  | **1** |  | **1** |  |  |  |  |  |  | **1** |  |  |  |  |  |  |  | **1** |  |  | **1** |  |  |  |  | **1** |  |  | **1** |
| 15 | **M15** | **1** |  |  | **1** | **1** |  |  | **1** | **1** | **1** |  |  |  |  |  | **1** |  |  |  |  |  |  |  |  | **1** |  |  | **1** |  |  |  |  | **1** |  | **1** |  |
| 16 | **M16** | **1** |  | **1** |  |  |  |  |  |  |  |  |  |  |  |  |  | **1** |  |  |  |  |  |  |  | **1** |  |  | **1** |  |  |  |  | **1** |  | **1** |  |
| 17 | **M17** | **1** |  | **1** |  |  |  |  |  |  |  |  |  |  |  |  | **1** |  |  |  |  |  |  |  | **1** |  |  |  | **1** |  |  | **1** |  |  |  | **1** |  |
| 18 | **M18** | **1** |  |  | **1** | **1** |  |  | **1** | **1** |  |  |  |  |  | **1** |  |  |  |  |  |  |  |  | **1** |  |  |  | **1** |  |  |  | **1** |  |  |  | **1** |
| 19 | **M19** | **1** |  | **1** |  |  |  |  |  |  |  |  |  |  |  |  |  | **1** |  |  |  |  |  |  |  | **1** |  |  | **1** |  |  | **1** |  |  |  |  | **1** |
| 20 | **M20** | **1** |  | **1** |  |  |  |  |  |  |  |  |  |  |  |  | **1** |  |  |  |  |  |  |  |  | **1** |  |  | **1** |  |  |  | **1** |  |  |  | **1** |
| 21 | **M21** | **1** |  | **1** |  |  |  |  |  |  |  |  |  |  |  |  | **1** |  |  |  |  |  |  |  |  | **1** |  |  | **1** |  |  |  |  | **1** |  |  | **1** |
| 22 | **WuFX-FIG3f** | **1** |  | **1** |  |  |  |  |  |  |  |  |  |  |  |  |  | **1** |  |  |  |  |  | **1** |  |  |  |  | **1** |  | **1** |  |  |  |  | **1** |  |
| 23 | **WuFX-FIG3i** |  | **1** |  | **1** | **1** |  |  | **1** | **1** | **1** |  |  |  |  |  | **1** |  |  |  |  |  |  |  | **1** |  | **1** |  |  |  | **1** |  |  |  |  | **1** |  |
| 24 | **WuFX-FIG3j** | **1** |  | **1** |  |  |  |  |  |  |  |  |  |  |  |  |  | **1** | **1** |  |  |  |  |  |  | **1** |  |  | **1** |  |  |  |  | **1** |  | **1** |  |

**Supplementary Table 3 │Climate-Leaf Analysis Multivariate Program (CLAMP) scoresheet for Jianglang (~47 Ma).** Colours of the heading line represent different leaf traits. Colours of scoring rows represent different leaf morphotypes.

| **Species  Number** | **Species / Morphotypes** | **Lamina** | | **Margin Character States** | | | | | | | | | | **Size Character States** | | | | | | | | | **Apex Character States** | | | | **Base Character States** | | | **Length to Width Character States** | | | | | **Shape Character States** | | |
| --- | --- | --- | --- | --- | --- | --- | --- | --- | --- | --- | --- | --- | --- | --- | --- | --- | --- | --- | --- | --- | --- | --- | --- | --- | --- | --- | --- | --- | --- | --- | --- | --- | --- | --- | --- | --- | --- |
|  |  | **Unlobed** | **Lobed** | **No Teeth** | **Teeth** | **Teeth Regular** | **Teeth Irregular** | **Teeth Close** | **Teeth Distant** | **Teeth Round** | **Teeth Acute** | **Teeth Compound** | **Compound<50%** | **Nanophyll** | **Leptophyll I** | **Leptophyll II** | **Microphyll I** | **Microphyll II** | **Microphyll III** | **Mesophyll I** | **Mesophyll II** | **Mesophyll III** | **Emarginate** | **Round** | **Acute** | **Attenuate** | **Cordate** | **Round** | **Acute** | **L:W <1:1** | **L:W 1-2:1** | **L:W 2-3:1** | **L:W 3-4:1** | **L:W >4:1** | **Obovate** | **Elliptic** | **Ovate** |
| 1 | **cf. Banksia** | **1** |  |  | **1** | **1** |  | **1** | **1** | **1** | **1** |  |  |  |  |  |  | **1** |  |  |  |  |  |  |  | **1** |  |  | **1** |  |  |  |  | **1** |  | **1** |  |
| 2 | **cf. Cardiospermum** | **1** |  |  | **1** |  | **1** |  | **1** |  | **1** |  |  |  |  | **1** | **1** |  |  |  |  |  |  |  | **1** |  |  |  | **1** |  |  | **1** | **1** |  |  | **1** |  |
| 3 | **Comptonia** |  | **1** | **1** |  |  |  |  |  |  |  |  |  | **1** |  |  |  |  |  |  |  |  |  |  |  |  |  |  |  |  |  |  |  | **1** |  | **1** |  |
| 4 | **Legume** | **1** |  | **1** |  |  |  |  |  |  |  |  |  |  |  | **1** |  |  |  |  |  |  |  | **1** |  |  |  |  | **1** |  |  |  |  | **1** |  | **1** |  |
| 5 | **Macclintockia** | **1** |  | **1** |  |  |  |  |  |  |  |  |  |  |  |  |  | **1** |  |  |  |  |  |  |  |  |  |  | **1** |  |  |  |  |  |  | **1** |  |
| 6 | **Menispermaceae** | **1** |  | **1** |  |  |  |  |  |  |  |  |  |  |  |  |  |  |  | **1** |  |  |  |  | **1** |  | **1** |  |  | **1** |  |  |  |  |  |  | **1** |
| 7 | **Syzygioides** | **1** |  | **1** |  |  |  |  |  |  |  |  |  |  |  |  | **1** |  |  |  |  |  |  |  |  | **1** |  |  | **1** |  |  |  |  | **1** |  | **1** |  |
| 8 | **JL5-Leaf-M01** | **1** |  | **1** |  |  |  |  |  |  |  |  |  |  |  |  | **1** |  |  |  |  |  |  |  | **1** |  |  |  | **1** |  |  | **1** |  |  |  | **1** |  |
| 9 | **JL5-Leaf-M02** | **1** |  | **1** | **1** | **1** |  |  | **1** |  | **1** |  |  |  |  |  |  | **1** |  |  |  |  |  |  |  |  |  |  | **1** |  |  |  |  | **1** |  | **1** |  |
| 10 | **JL5-Leaf-M03** | **1** |  | **1** |  |  |  |  |  |  |  |  |  |  |  |  | **1** |  |  |  |  |  |  |  | **1** |  |  |  | **1** |  |  |  |  | **1** |  | **1** |  |
| 11 | **Cedrelospermum**  **(Ulmaceae) leaf** | **1** |  |  | **1** | **1** |  | **1** |  | **1** |  | **1** | **1** |  |  |  | **1** | **1** |  |  |  |  |  |  |  | **1** |  | **1** | **1** |  |  | **1** | **1** |  |  | **1** |  |
| 12 | **JL5-Leaf-M05** |  | **1** | **1** |  |  |  |  |  |  |  |  |  |  | **1** | **1** |  |  |  |  |  |  |  | **1** |  | **1** |  |  | **1** |  |  | **1** | **1** |  |  |  | **1** |
| 13 | **JL5-Leaf-M06** | **1** |  | **1** |  |  |  |  |  |  |  |  |  |  |  |  | **1** |  |  |  |  |  |  | **1** |  |  |  |  | **1** |  |  |  | **1** |  |  | **1** |  |
| 14 | **Myrtales leaf** | **1** |  | **1** |  |  |  |  |  |  |  |  |  |  |  |  |  | **1** |  |  |  |  |  |  |  |  |  |  | **1** |  |  |  |  | **1** |  | **1** |  |
| 15 | **JL5-Leaf-M08** | **1** |  |  | **1** | **1** |  | **1** |  | **1** |  |  |  |  |  |  |  | **1** |  |  |  |  |  |  |  |  |  |  | **1** |  | **1** |  |  |  |  | **1** |  |
| 16 | **JL5-Leaf-M09** | **1** |  | **1** |  |  |  |  |  |  |  |  |  |  |  |  | **1** |  |  |  |  |  |  |  |  |  |  |  | **1** |  |  | **1** |  |  |  | **1** |  |
| 17 | **JL5-Leaf-M10** | **1** |  |  | **1** |  | **1** |  | **1** | **1** |  |  |  |  | **1** |  |  |  |  |  |  |  |  | **1** |  |  |  |  | **1** |  | **1** |  |  |  |  | **1** |  |
| 18 | **JL5-Leaf-M11** | **1** |  | **1** |  |  |  |  |  |  |  |  |  |  |  |  |  |  | **1** |  |  |  |  |  |  | **1** |  |  | **1** |  |  |  |  | **1** |  |  | **1** |
| 19 | **JL5-Leaf-M12** | **1** |  | **1** |  |  |  |  |  |  |  |  |  |  |  |  |  |  | **1** | **1** |  |  |  |  |  | **1** |  | **1** | **1** |  |  |  | **1** | **1** |  | **1** |  |
| 20 | **JL5-Leaf-M13** | **1** |  | **1** |  |  |  |  |  |  |  |  |  |  |  |  |  |  |  | **1** |  |  |  |  |  | **1** |  |  | **1** |  |  | **1** |  |  |  | **1** |  |
| 21 | **JL5-Leaf-M14** | **1** |  |  | **1** | **1** |  |  | **1** |  | **1** |  |  |  |  |  |  |  |  | **1** |  |  |  |  |  | **1** |  |  | **1** |  |  |  | **1** |  |  | **1** |  |
| 22 | **JL5-Leaf-M15** | **1** |  | **1** |  |  |  |  |  |  |  |  |  |  |  |  |  |  |  | **1** |  |  |  |  |  |  |  |  |  |  |  |  |  |  |  | **1** |  |
| 23 | **JL5-Leaf-M16** | **1** |  | **1** |  |  |  |  |  |  |  |  |  |  |  |  |  | **1** | **1** |  |  |  |  |  |  |  |  |  | **1** |  |  |  |  | **1** |  | **1** |  |
| 24 | **JL5-Leaf-M17** | **1** |  |  | **1** | **1** |  |  | **1** |  | **1** |  |  |  |  |  |  | **1** |  |  |  |  |  |  |  | **1** |  |  | **1** |  |  |  |  | **1** |  | **1** |  |
| 25 | **JL5-Leaf-M18** | **1** |  | **1** |  |  |  |  |  |  |  |  |  |  |  |  |  | **1** |  |  |  |  |  |  |  | **1** |  |  | **1** |  |  |  |  | **1** |  | **1** |  |
| 26 | **JL5-Leaf-M19** | **1** |  | **1** |  |  |  |  |  |  |  |  |  |  |  |  |  | **1** |  |  |  |  |  |  |  | **1** |  |  | **1** |  |  |  |  | **1** |  | **1** |  |
| 27 | **JL5-Leaf-M20** | **1** |  |  | **1** | **1** |  |  | **1** |  | **1** |  |  |  |  |  |  | **1** |  |  |  |  |  |  |  |  |  |  | **1** |  |  |  |  | **1** |  | **1** |  |
| 28 | **JL5-Leaf-M21** | **1** |  | **1** |  |  |  |  |  |  |  |  |  |  |  |  | **1** | **1** |  |  |  |  |  |  | **1** |  |  |  | **1** |  |  |  |  | **1** |  | **1** |  |
| 29 | **JL5-Leaf-M04** | **1** |  | **1** |  |  |  |  |  |  |  |  |  |  |  |  |  | **1** |  |  |  |  |  |  |  |  |  |  | **1** |  |  |  |  |  |  | **1** |  |
| 30 | **JL5-Leaf-M07** | **1** |  | **1** |  |  |  |  |  |  |  |  |  |  |  |  |  |  | **1** |  |  |  |  |  |  |  |  |  | **1** |  |  |  |  | **1** |  | **1** |  |
| 31 | **cf. Ailanthus**  **(Simaroubaceae) leaf** | **1** |  | **1** |  |  |  |  |  |  |  |  |  |  |  |  |  |  |  | **1** |  |  |  |  |  |  | **1** |  |  |  |  | **1** |  |  |  |  | **1** |

**Supplementary Table 4 │Phytolith statistics of the Dayu and Jianglang sections.**

| Sample number | Non-diagnostic "grass" phytoliths (NDG) | Non-diagnostic and unclassified phytoliths (NDO) | Total phytoliths counted | FI-t ratio | Forest indicator taxa (FI TOT) | | | | | | Grass short cell silica (GSSC) | | | | Other biosilica | |
| --- | --- | --- | --- | --- | --- | --- | --- | --- | --- | --- | --- | --- | --- | --- | --- | --- |
|  |  |  |  | (FI TOT)/  (FITOT+GSSC) % | Globular | Polyhedral/ anticlinal epidermis | Blocky polyhedron | Sclerenchyma and similiar | Irregular vascular cell | Vesicular infilling | Rondel | Bilobate | Saddle | GSSC bulliform | Diatoms | Sponge spicules |
| JL-4 | - | 6 | 22 | - | 1 | 7 | 12 | - | 2 | - | - | - | - | - | - | - |
| DY1-2 | 7 | 17 | 130 | 94.6 | - | 60 | 52 | - | 8 | 3 | - | - | - | 7 | - | - |
| DY1-15 | 8 | 41 | 110 | 85.8 | - | 59 | 35 | 2 | 7 | - | - | - | - | 7 | - | - |
| DY1-71 | 6 | 23 | 50 | 94.0 | - | 9 | 38 | - | - | - | - | 1 | 0 | 2 | - | - |
| DY-Fossil-bearing layer | 12 | 78 | 83 | 33.7 | - | - | 26 | 2 | - | - | 5 | - | 4 | 46 | 237 | 5 |

**Supplementary Table 5 │Modern affinities of elements of the Dayu flora that potentially have spines.**

| **Morphotype** | **Family/Order** | **Presence of spiny species** | **Notes** |
| --- | --- | --- | --- |
| M01 | Malvaceae | yes | morphotype 1 |
| M02 | Cedrelospermum | yes |  |
| M03 | Cannabaceae | yes |  |
| M04 | Desmodium | yes |  |
| M05 | Rosales | yes |  |
| M06 | Ulmus | yes |  |
| M07 | undetermined |  | Small and lobed leaf |
| M08 | Menispermaceae | yes |  |
| M09 | Ailanthus | yes |  |
| M10 | Anacardiaceae | yes |  |
| M11 | undetermined |  | Leaf base asymmetric with apex attenuate |
| M12 | Myrtaceae | yes |  |
| M13 | Malvaceae | yes | morphotype 2 |
| M14 | Syzygium | yes |  |
| M15 | undetermined |  | Leaf elongate with regular marginal teeth |
| M16 | undetermined |  | Leaf base acute with margin entire |
| M17 | Fabaceae | yes | morphotype 1 |
| M18 | undetermined |  | Small leaf with few teeth |
| M19 | undetermined |  | Small and elliptic leaf with entire margin |
| M20 | Fabaceae | yes | morphotype 2 |
| M21 | Pistacia | yes |  |
| WuFX-FIG3f | undetermined |  | Small leaf with round apex and acute base |
| WuFX-FIG3i | Araliaceae | yes |  |
| WuFX-FIG3j | Handeliodendron | no |  |
| Notes: WuFX-FIG3f, WuFX-FIG3i, WuFX-FIG3j are from Wu et al., 2017, Scientific Reports \| 7: 878 \| DOI:10.1038/s41598-017-00928-9 | | | |

**Supplementary Note 1 │****Detailed morphological description of each morphotype of fossil spines.**

Spiny fossils in this study were divided into prickles and thorns. Prickles originate from plant epidermal tissue and can be easily detached from stems. They are usually randomly distributed on the stems. Since there exists an abscission layer where prickles attach to the stem, prickles tend to detach from the stem when they are fossilized, but they leave a distinct scar at the junction between the prickles and stems. This shows the prickle is not attached to the vascular bundle, which is a key morphological feature distinguishing prickles from thorns. Some fossils of individual prickles were also found in the same layer. Thorns originate from vascular bundles and are not easily broken or detached from stems. They are usually longer than prickles and grow on the stem regularly. On the fossils, the thorns connect to the stem without scars. According to size, growth pattern, and density, we further classified prickles into two types and thorns into five types.

1. Prickles

1.1 Morphotype Ⅰ: Prickles grow on stems alternately. The average length of prickles is 4.9 ± 1.6 mm. The mean width at base of each prickle is 5.8 ± 1.2 mm. Prickles curve upward at an angle of ~60°.

1.2 Morphotype Ⅱ: Prickles occur on stems irregularly. The average length of prickles is 3.8 ± 1.2 mm. The mean width at base of prickles is 5.2 ± 1.5 mm. Prickles grow on stems at an angle of ~90°.

2. Thorns

2.1 Morphotype Ⅲ: Thorns grow on stems oppositely. The branch is 12.15 cm long, and densely covered with 13 thorns. The average length of thorns is 5.6 ± 2.1 mm. Thorns grow on stems at an angle of ~90°.

2.2 Morphotype Ⅳ: Thorns grow on stems alternately. The average length of thorns is 4.7 ± 1.7 mm. Each thorn curves upward or downward.

2.3 Morphotype Ⅴ: Thorns grow on stems alternately. The average length of thorns is 29.0 ± 14.7 mm. They grow on the stem at an angle of ~90°.

2.4 Morphotype Ⅵ: Thorns grow on stems alternately in an angle less than 45°. The average length of thorns is 5.9 ± 1.4 mm.

2.5 Morphotype Ⅶ: Thorns grow on stems oppositely and in pairs. The average length of thorns is 2.6 ± 0.6 mm. They grow on the stems at an angle of ~80°.

**Supplementary Note 2 │Palaeoelevation reconstruction of Dayu at ~39 Ma.**

Climate-Leaf Analysis Multivariate Program (CLAMP) derives moist enthalpy from leaf form and was previously applied to the Jianglang flora (327 kJ/kg, Supplementary Table 3) and using climate model output moderated by sea level CLAMP data the height of the Jianglang flora was determined to be 1.5 ± 0.9 km^4^. The CLAMP-derived moist enthalpy for the Dayu flora is 316 kJ/kg (Supplementary Table 1) so the height difference between the Jianglang and Dayu flora is

(327 – 316)/9.81 = 1.1 km (1)

making the absolute elevation of the Dayu flora

1.5 + 1.1 = 2.6 ± 1.2 km (2)

The uncertainty is determined by square root of the sum of the squared uncertainties (0.9 km) for each site.

This approach ignores the age difference (5–7 Ma) between the Jianglang and Dayu floras and any change in the secular climate-driven moist enthalpy regime. We assume that this difference will be minimal over that interval and at these latitudes given what we know about global climate change between 47 Ma and 38 Ma^5^, but here we also use wet bulb terrestrial lapse rates for verification.

In addition to using moist enthalpy, palaeoelevation can also be derived using local terrestrial thermal lapse rates, and recently it has been shown that the most robust way of doing this is by using wet bulb temperatures instead of the normal dry bulb measures^6^.

We applied CLAMP to the early Eocene sea level leaf assemblage from the Gurha Mine in north western India^1^ to derive wet bulb mean annual temperature at sea level (MAT_wsl_) (21 °C). This site provided the sea level datum for the Su et al. (2020)^4^ analysis of the Jianglang elevation and was adjusted using a climate model for the palaeolatitudinal difference between Gurha and the Lunpola Basin. This approach gave an estimate of MAT_wsl_ for Dayu of 20.5 °C. Using a climate model with Priabonian boundary conditions and a nominal central Tibetan valley system where the valley floor was set to 2.5 km above sea level, we derive a mean annual *Γε* of 3.821 °C/km. Using this mean annual *Γε* the difference in MAT_wsl_ and MAT_wD_ at Dayu gives the height of Dayu as:

20.5 - 10.2 = 2.7 ± 0.9 km (3)

Uncertainties are given by the square root of the sum of the squared statistical uncertainty of deriving MAT_wsl_ and MAT_wD_ from CLAMP (2.5 °C in both cases) divided by *Γε*. This does not include model uncertainties, which are hard to quantify.

Both the moist enthalpy and the wet bulb terrestrial lapse rate approaches give similar results for the elevation of the Dayu fossil assemblage: 2.6 ± 1.2 km and 2.7 ± 0.9 km respectively.

**Supplementary Note 3 │Reconstructed vegetation using climate modelling.**

To objectively reconstruct Eocene vegetation types within the Lunpola region we used output from the same fully coupled Atmosphere-Ocean General Circulation Model (AOGCM), HadCM3BL-M2.1Ad^7^ as was used to generate wet-bulb terrestrial lapse rates within a Tibetan valley system (Supplementary Figure 14A). The predicted climate, including moisture conditions down to a soil depth of 65 cm, input to the Triffid vegetation model^8,9^ generated a varying mix of bare soil, some C_3_ ground cover, some broadleaved taxa, some shrubs, and a predominance of needle-leaved tree taxa, which includes not just conifers but any trees with small leaves (Supplementary Figure 14B). This prediction is entirely consistent with open woodland vegetation adapted to seasonal drought. Precipitation and soil moisture metrics pointed to a winter-wet regime at the western end of the valley system drying to the east, where some rainfall also occurred resulting in a bimodal rainfall pattern in an overall semi-arid climate (Supplementary Figure 14C).

**Supplementary References**

1. Wu, F. X., Miao, D. S., Chang, M. M., Shi, G. L. & Wang, N. Fossil climbing perch and associated plant megafossils indicate a warm and wet central Tibet during the late Oligocene. *Sci. Rep.* **7,** 878 (2017).

2. Lu, H. Y. *et al*. Phytoliths as quantitative indicators for the reconstruction of past environmental conditions in China II: palaeoenvironmental reconstruction in the Loess Plateau. *Quat. Sci. Rev.* **25,** 945-959 (2006).

3. Su, T. *et al.* No high Tibetan Plateau until the Neogene. *Sci. Adv.* **5,** eaav2189, (2019).

4. Su, T. *et al.* A Middle Eocene lowland humid subtropical “Shangri-La” ecosystem in central Tibet. *Proc. Natl Acad. Sci. USA* **117,** 32989-32995 (2020).

5. Westerhold, T. *et al.* An astronomically dated record of Earth’s climate and its predictability over the last 66 million years. *Science* **369,** 1383-1387 (2020).

6. Farnsworth, A. *et al.* Paleoclimate model-derived thermal lapse rates: Towards increasing precision in paleoaltimetry studies. *Earth Planet. Sci. Lett.* **564,** 116903 (2021).

7. Valdes, P. J. et al. The BRIDGE HadCM3 family of climate models: HadCM3@ Bristol v1.0. *Geosci. Model Dev.* **10,** 3715-3743 (2017).

8. Cox, P. M. Description of the “TRIFFID” dynamic global vegetation model. 1-16 (Met Office Hadley Centre 2001).

9. Cox, P., Huntingford, C. & Harding, R. A canopy conductance and photosynthesis model for use in a GCM land surface scheme. *J. Hydrol.* **212,** 79-94 (1998).
