# Supplementary material for: Rapid Eocene diversification of spiny plants in subtropical woodlands of central Tibet
Source: Nat Commun. 2022 Jul 1;13:3787. doi: 10.1038/s41467-022-31512-z (PMC9249787; doi:10.1038/s41467-022-31512-z)
Supplement: Supplementary file 3 — Description of Additional Supplementary Files [file 41467_2022_31512_MOESM3_ESM.pdf]

Description of Additional Supplementary Files

File Name: Supplementary Data 1
Description: Paleogene records of herbivorous mammals at genus level on the Tibetan Plateau and regions nearby.

File Name: Supplementary Code 1
Description: Code for phylogeny reconstruction of spiny plants in eudicots in Eurasia during the Cenozoic. Supplementary References
