# Supplementary material for: Rapid Eocene diversification of spiny plants in subtropical woodlands of central Tibet
Source: Nat Commun. 2022 Jul 1;13:3787. doi: 10.1038/s41467-022-31512-z (PMC9249787; doi:10.1038/s41467-022-31512-z)
Supplement: Supplementary file 5 — Supplementary Code 1 [file 41467_2022_31512_MOESM5_ESM.docx]

**Supplementary Code 1 for ‘****Rapid Eocene diversification of spiny plants in subtropical woodlands of central Tibet’**

**Code for phylogeny reconstruction of spiny plants in eudicots in Eurasia during the Cenozoic.** R version 4.0.5 with library phytools^1^, ape^2^ and Geiger^3^ were used for phylogenetic analyses.

library(ape)

library(geiger)

library(phytools)

tree<-read.tree("/woody_eudicot_tree.tre")

data<-read.csv("/woody_eudicot_data.csv")

orderedtaxa=tree$tip.label

#check if names in phylogeny and data match

data <- data[match(orderedtaxa, data$Genus_Species), ]

#get list of continents in data set

continent<-data$continent

names(continent)<-data$Genus_Species

#extract the spines data

spines<-(data$spinystem)###added +1

names(spines)<-data$Genus_Species

#Run for a given continent

cont.name<-as.character(unique(data$continent))

cont.name

#######################################

#ASIA

#prune the dataset and the tree to species in Asia

continentASIA<-subset(data, continent == "Asia")

rownames=continentASIA$Genus_Species

continent.treeASIA<-drop.tip(tree, tree$tip.label[!tree$tip.label %in% as.vector(continentASIA$Genus_Species)])

continent.spinesASIA<-(continentASIA$spinystem)

names(continent.spinesASIA)<-continentASIA$Genus_Species

#extract the spines data

spincontAsia<-setNames(as.factor(continent.spinesASIA),continentASIA$Genus_Species)

summary(spincontAsia)

#define irreversible model

model<-matrix(c(0,0,1,0),2,2)

#Fit model in Geiger

fitIrrdeltaASIA<-fitDiscrete(continent.treeASIA, spincontAsia, model =model, transform = c("delta"), bounds = list(delta = c(min = 0, max = 60)), control=list(niter=100))

#extract delta form the fitted model and rescale tree

my.deltaASIA<-fitIrrdeltaASIA$opt$delta

delta.treeASIA<-rescale(continent.treeASIA, "delta", my.deltaASIA)

#use stochastic character mapping to map traits on the rescaled tree using phytools

Nsims<-100

spine.treesASIAdelta<-make.simmap(delta.treeASIA,continent.spinesASIA,model=model, pi = c(1,0), nsim=Nsims)#

# function to compute the states at each node in the tree - this comes form Liam Revel:

#http://blog.phytools.org/2013/03/new-totally-rewritten-version-of.html

foo<-function(x){

y<-sapply(x$maps,function(x) names(x)[1])

names(y)<-x$edge[,1]

y<-y[as.character(length(x$tip)+1:x$Nnode)]

return(y)

}

#Run function to calculate ancestral states at each node

AAASIA<-sapply(spine.treesASIAdelta,foo)

#calculate node ages

agesASIA<-branching.times(continent.treeASIA)

#Run across nodes and calculate proportion of stochastic character mapped states in each state for each node

transitionsASIA<-NA

for (n in 1:length(agesASIA)){

z<-AAASIA[n,]

zz<-z[z == "1"]##

transitionsASIA[n]<-length(zz)/Nsims

}

###to calculate proportion

transitionsNOASIA<-NA

for (n in 1:length(agesASIA)){

z<-AAASIA[n,]

zz<-z[z == "0"]##

transitionsNOASIA[n]<-length(zz)/Nsims

}

##Spiny

#join node ages and ancestral state data

anc.outputASIA<-cbind(agesASIA, transitionsASIA)

#sort database by node age (oldest node first)

ordered.anc.outputASIA<-anc.outputASIA[order (-agesASIA),]

##Non-spiny

#join node ages and ancestral state data

anc.outputNOASIA<-cbind(agesASIA, transitionsNOASIA)

#sort database by node age (oldest node first)

ordered.anc.outputNOASIA<-anc.outputNOASIA[order (-agesASIA),]

#calculate spiny lineages through time

#here sum of lineages weighted for the probability of being spiny oppose to imposing some arbitrary threshold

##Spiny

asia.spines<-NULL

spine.counter<-0

for (k in 1:length(agesASIA)) {

spine.counter<-spine.counter+ordered.anc.outputASIA[k,2]

asia.spines[k]<-spine.counter

}

##Non-spiny

asia.NOspines<-NULL

NOspine.counter<-0

for (k in 1:length(agesASIA)) {

NOspine.counter<-NOspine.counter+ordered.anc.outputNOASIA[k,2]

asia.NOspines[k]<-NOspine.counter

}

#new data form with node ages and number of spiny lineages

resultsASIA<-as.data.frame(cbind(ordered.anc.outputASIA[,"agesASIA"], asia.spines, asia.NOspines))

colnames(resultsASIA)[1]<-"agesASIA"

###log cumulative plot in figure 3

plot(resultsASIA$agesASIA, log(resultsASIA$asia.spines/sum(resultsASIA$asia.NOspines+resultsASIA$asia.spines)), type = "l", col = "red", xlim = c(65, min(resultsASIA$ages)), xlab = "Ma", ylab = "lineages", main = "Asia_spinification_0.103")

**Supplementary References**

1. Revell, L. J. phytools: an R package for phylogenetic comparative biology (and other things). *Methods Ecol. Evol*. **3,** 217-223 (2012).

2. Paradis, E. & Schliep, K. ape 5.0: an environment for modern phylogenetics and evolutionary analyses in R. *Bioinformatics* **35,** 526-528 (2019).

3. Harmon, L. J., Weir, J. T., Brock, C. D., Glor, R. E. & Challenger, W. GEIGER: investigating evolutionary radiations. *Bioinformatics* **24,** 129-131 (2008).
