# Supplementary material for: Rapid Eocene diversification of spiny plants in subtropical woodlands of central Tibet
Source: Nat Commun. 2022 Jul 1;13:3787. doi: 10.1038/s41467-022-31512-z (PMC9249787; doi:10.1038/s41467-022-31512-z)
Supplement: Supplementary file 6 — Reporting Summary [file 41467_2022_31512_MOESM6_ESM.pdf]

nature portfolio

Corresponding author(s): Su Tao
Last updated by author(s): Jun 16, 2022

Reporting Summary

Nature Portfolio wishes to improve the reproducibility of the work that we publish. This form provides structure for consistency and transparency in reporting. For further information on Nature Portfolio policies, see our Editorial Policies and the Editorial Policy Checklist.

Statistics

For all statistical analyses, confirm that the following items are present in the figure legend, table legend, main text, or Methods section.

n/a	Confirmed	
☐	☒	The exact sample size (n) for each experimental group/condition, given as a discrete number and unit of measurement
☐	☒	A statement on whether measurements were taken from distinct samples or whether the same sample was measured repeatedly
☒	☐	The statistical test(s) used AND whether they are one- or two-sided<br>*Only common tests should be described solely by name; describe more complex techniques in the Methods section.*
☐	☒	A description of all covariates tested
☐	☒	A description of any assumptions or corrections, such as tests of normality and adjustment for multiple comparisons
☐	☒	A full description of the statistical parameters including central tendency (e.g. means) or other basic estimates (e.g. regression coefficient) AND variation (e.g. standard deviation) or associated estimates of uncertainty (e.g. confidence intervals)
☒	☐	For null hypothesis testing, the test statistic (e.g. F, t, r) with confidence intervals, effect sizes, degrees of freedom and P value noted<br>*Give P values as exact values whenever suitable.*
☒	☐	For Bayesian analysis, information on the choice of priors and Markov chain Monte Carlo settings
☐	☒	For hierarchical and complex designs, identification of the appropriate level for tests and full reporting of outcomes
☒	☐	Estimates of effect sizes (e.g. Cohen's d, Pearson's r), indicating how they were calculated

Our web collection on statistics for biologists contains articles on many of the points above.

Software and code

Policy information about availability of computer code

Data collection	No software was used when collecting data.
Data analysis	We used the following softwares in the study: QGIS version 3.16 for location marking, ImageJ version 1.52a for size measurement, and R version 4.0.5 with library "phytools 1.0-3", "ape 5.6-2" and "Geiger 2.0.9" for phylogenetic analyses.<br>We use methods including Climate-Leaf Analysis Multivariate Program (CLAMP) (http://clamp.ibcas.ac.cn) for palaeoenvironmental reconstructions, and Atmosphere-Ocean General Circulation Model (AOGCM), HadCM3BL-M2.1aD for palaeovegetation predictions. Model data can be accessed at http://bridge.bris.ac.uk/resources/simulations.

For manuscripts utilizing custom algorithms or software that are central to the research but not yet described in published literature, software must be made available to editors and reviewers. We strongly encourage code deposition in a community repository (e.g. GitHub). See the Nature Portfolio guidelines for submitting code & software for further information.

Data

Policy information about availability of data

All manuscripts must include a data availability statement. This statement should provide the following information, where applicable:

- Accession codes, unique identifiers, or web links for publicly available datasets
- A description of any restrictions on data availability
- For clinical datasets or third party data, please ensure that the statement adheres to our policy

All data analyzed in this paper are available as parts of the article and supplementary files.
Woody spiny eudicots in Eurasia are from JSTOR Global Plants (https://plants.jstor.org/).
Fossil records of Paleogene herbivorous mammals are from fossilworks (http://fossilworks.org/).

Climate and vegetation simulation data are accessed at http://bridge.bris.ac.uk/resources/simulations.
The PhysgAsia2 Calibration Files are accessed at http://clamp.ibcas.ac.cn/CLAMP_PhysgAsia2.html.
The Worldclime Version2 climate data are accessed at http://worldclim.com/version2.

Field-specific reporting

Please select the one below that is the best fit for your research. If you are not sure, read the appropriate sections before making your selection.

☐ Life sciences ☐ Behavioural & social sciences ☒ Ecological, evolutionary & environmental sciences

For a reference copy of the document with all sections, see nature.com/documents/nr-reporting-summary-flat.pdf

Ecological, evolutionary & environmental sciences study design

All studies must disclose on these points even when the disclosure is negative.

Study description	This is a palaeontological and palaeoecology study including collection, digital imaging, description of spiny plant fossil materials from middle Eocene sediments of central Tibetan Plateau. Analyses including phylogeny, phytoliths, grass fossils, modeling and mammal fossils, are used to explore the diversification history of plant spines in Asia.
Research sample	A total of 44 specimens of plant spine fossils, all with sharp spines and regularly or randomly distributed on the branches. We chose them for morphological analysis because they are well-preserved and have clear identification characteristics. These materials derive from the grayish green mudstones and sandstones of the Niubao Formation (Bartonian, early late Eocene) in Lunpola Basin, Tibet, China.
Sampling strategy	We collected more than 1000 plant fossil specimens from the Dayu and Xiede sections, among them, 44 well-preserved specimens of plant spine fossils were found and used in this study.
Data collection	Professor Tao Su led all the field expeditions in Lunpola Basin, central Tibetan Plateau from July 2016 to August 2020. The digital images of specimens were collected using Nikon digital camera and a stereoscope in Public Technology Service Center of Xishuangbanna Tropical Botanical Garden. Measurements of the length, width and angle of plant fossils were performed using digital images of fossil specimens.
Timing and spatial scale	We collected plant fossils from 2016 to 2020 in Dayu and Xiede sections, which belong to the Niubao Formation and are located in Lunpola Basin, central Tibetan Plateau. We collected fossil specimens with colleagues from Xishuangbanna Tropical Botanical Garden and Institute of Vertebrate Paleontology and Paleoanthropology, as well as local Tibetan villagers.
Data exclusions	No data are excluded.
Reproducibility	Three replicate experiments of phytolith analysis were carried out on the sediments of the Dayu section. Each repetition of the experiment yielded similar results, with consistent diversity and number of phytoliths.
Randomization	No randomization procedure was used because it was not necessary for the type of study we conducted.
Blinding	This is a palaeontological study of all known fossil materials. Blinding is inapplicable and irrelevant.

Did the study involve field work? ☒ Yes ☐ No

Field work, collection and transport

Field conditions	Middle Eocene grayish green mudstones and sandstones are well-exposed in both fossil sites, fossils were collected directly from these rocks. The average elevation in central Tibetan Plateau is about 4,700 meters, dominated by alpine steppe, with mean annual temperature of 0–3°C and mean annual precipitation of ~300 mm.
Location	There are two fossil sites in this study, and both are located in central Tibetan Plateau, China.<br>1. Dayu section: 32°20′N, 89°46′E, 4700m;<br>2. Xiede section: 31°58′N, 88°25′E, 4655m.
Access & import/export	All fossil specimens in this study were collected by paleontological expedition team from Xishuangbanna Tropical Botanical Garden and Institute of Vertebrate Paleontology and Paleoanthropology in compliance with all local, national and international laws. All collecting permissions were obtained before the field work.
Disturbance	All samples were collected by ourselves and local Tibetan people without disturbance of the surrounding environment.

Reporting for specific materials, systems and methods

We require information from authors about some types of materials, experimental systems and methods used in many studies. Here, indicate whether each material, system or method listed is relevant to your study. If you are not sure if a list item applies to your research, read the appropriate section before selecting a response.

Materials & experimental systems

n/a	Involved in the study
☒	☐ Antibodies
☒	☐ Eukaryotic cell lines
☐	☒ Palaeontology and archaeology
☒	☐ Animals and other organisms
☒	☐ Human research participants
☒	☐ Clinical data
☒	☐ Dual use research of concern

Methods

n/a	Involved in the study
☒	☐ ChIP-seq
☒	☐ Flow cytometry
☒	☐ MRI-based neuroimaging

Palaeontology and Archaeology

Specimen provenance

Specimens were collected by ourselves from the Dayu and Xiede sections along the Bangong-Nujiang Suture Zone, central Tibet, China. The permits were obtained by local governments in Xizang Autonomous Region, China. The fossil excavation was permitted by Department of Natural Resources and Department of Science & Technology in Tibetan Autonomous Region, China.

Specimen deposition

All fossil specimens are deposited at Paleoecological Collections, Xishuangbanna Tropical Botanical Garden, Chinese Academy of Sciences.

Dating methods

Recently, tuff was discovered from the same fossil site as spiny fossils in the Dayu section, radiometric dating was carried out by U-Pb dating using zircons (Xiong et al., 2022, Science Advances, 8, eabj0944). They found three tuff samples within the middle member of the Niubao Formation in the Dayu section, and the age is constrained to range from 40 to 38 Ma. They carried out the experiment at the State Key Laboratory of Tibetan Plateau Earth System, Environment and Resources (TPESER), Institute of Tibetan Plateau Research, Chinese Academy of Sciences (ITPCAS). We cited their dating results in this study.

☒ Tick this box to confirm that the raw and calibrated dates are available in the paper or in Supplementary Information.

Ethics oversight

No ethics permissions are required to undertake our study because these specimens are fossil specimens and no live material was used.

Note that full information on the approval of the study protocol must also be provided in the manuscript.
